# *Immunity & Ageing*: a new journal looking at ageing from an immunological point of view

**DOI:** 10.1186/1742-4933-1-1

**Published:** 2004-10-29

**Authors:** Sonya Vasto, Calogero Caruso

**Affiliations:** 1 Managing Editor Gruppo di Studio sull'Immunosenescenza, Dipartimento di Biopatologia e Metodologie Biomediche, Università di Palermo, Corso Tukory 211, 90134 Palermo, Italy; 2 Editor-in-Chief Dipartimento di Biopatologia e Metodologie Biomediche, Università di Palermo, Corso Tukory 211, 90134 Palermo, Italy

**Keywords:** ageing, immunosenescence, inflammation, open access

## Abstract

In the elderly, many alterations of both innate and clonotypic immunity have been described. Alterations to the immune system in the elderly are generally viewed as a deterioration of immunity, leading to the use of the term immunosenescence. However, although many immunological parameters are often notably reduced in the elderly, retained function of both innate and clonotypic immunity in the elderly is tightly correlated to health status. Recognising the important role of the immune system in ageing, over the last few years, journals oriented towards gerontology and geriatric sciences have increasingly published articles dealing with the immunology of ageing, but a specialised journal in this area does not exist. *Immunity & Ageing *is a new Open Access, peer reviewed journal that aims to cover all the topics dealing with innate and clonotypic immunity which are relevant to ageing. The journal will provide an opportunity to focus on this topic, which is emerging as one of the critical mechanisms of ageing. Furthermore, as an online, Open Access journal, *Immunity & Ageing *will promote immediate accessibility to research, which is generally not possible for articles published in printed journals. We hope this forum, concentrating on the themes of ageing and immunology with a strong focus on human studies, will create a new perspective for viewing a world that is inevitably becoming older.

## 

*Immunity & Ageing *is a new Open Access, peer reviewed journal that aims to provide a forum for articles examining ageing from an immunological point of view.

During the past century, humans have gained more years of average life expectancy than in the last 10,000 years; we are now living in a rapidly ageing world. The sharp rise in life expectancy, coupled to a steady decline in birth rates in all developed countries, has led to an unprecedented demographic revolution characterized by an explosive growth in the number and proportion of older people. The number of people aged 60 years or older exceeded 635 million in 2002, and is expected to grow to nearly 2 billion by 2050. The proportion of people aged 60 and over stands about 1 in 4 in many Western European countries as well as in Japan. Should the present trend continues, this ratio is expected to reach 1 in 3 by 2050 [1]. Among the aged, the oldest old (>85) make up the fastest growing category. As access to medical care improves worldwide, the rate of population ageing will accelerate. If global communications is making the world "young and fast", then global ageing is surely "maturing and slowing" it. In any case, these epidemiological facts underscore the importance of studies on successful and unsuccessful ageing and necessitate the prompt spread of knowledge about ageing in order to satisfactorily decrease the medical, economic and social problems associated with advancing years.

## Ageing

Ageing is a post-maturational process that, due to a diminished homeostatic capacity and increased vulnerability, reduces responsiveness to environmental stimuli and is generally associated with an increased predisposition to illness and death. At the beginning of the 19^th ^century, mortality was described as increasing exponentially with respect to progression through the lifespan [2]. This trend, also described in invertebrates, persists: in Western countries the mortality rate increases 25 times more rapidly in individuals over 60 years old compared to people aged 25–44. Causes of death in aged people are increased compared with individuals between 25 and 44 years old: cancer 43-fold, pneumonia and influenza 89-fold, heart disease 92-fold and stroke and chronic lung disease greater than 100-fold [3]. Thus far, to understand ageing mechanisms, much attention has been paid to gene mutations in invertebrates and caloric restriction in rodents. However, these data suggest a key role for immunity in the survival of the elderly because susceptibility to these diseases depends at least in part on optimal immune function [4,5]. So, a better understanding of the ageing immune system may provide the most important clues for slowing the inevitable decline associated with the passage of time.

## Immunity in ageing

In the elderly, many alterations in innate and clonotypic immunity have been described and viewed as deleterious, hence the term immunosenescence. In 1969, Roy Walford published his landmark book, "The Immunologic Theory of Aging", and first coined the term immunosenescence [6]. Significantly, most of the areas that he pioneered during his illustrious research career remain the "hot" areas of current gerontological research. On the other hand, immunosenescence is a complex process involving multiple reorganizational and developmentally regulated changes, rather than simple unidirectional decline of the whole function [7,8]. However, some immunological parameters are commonly notably reduced in the elderly and, reciprocally good function is tightly correlated to health status [4,5].

## Innate immunity in ageing

The process of maintaining life for the individual is a constant struggle to preserve his/her integrity. This can come at a price when immunity is involved, namely systemic inflammation [9]. Inflammation is not a negative phenomenon per se: it is the response of the immune system to the invasion of viruses or bacteria and other pathogens. The problem is that in the course of evolution the human organism was set to live 40 or 50 years. Today, however, the immune system must remain active for longer. This very long activity leads to a chronic inflammation that slowly but inexorably damages all the organs: this is the typical phenomenon linked to ageing and it is considered the major risk factor for age-related chronic diseases, such as osteoporosis, sarcopenia, type 2 diabetes, Alzheimer's disease and atherosclerosis, though progression seems also dependent on the genetic background of individuals [4,5,8,10,11]. Emerging evidence suggests that pro-inflammatory genotypes are related to unsuccessful ageing, and, reciprocally, controlling inflammatory status may allow a better chance of successful ageing [4,5,8,12]. In other words, age-related diseases are "the price we pay" for an active immune system that defends us thorough out life, but also has the capacity to harm us later, as its fine tuning becomes compromised [13]. In fact, our immune system has evolved to control pathogens, so pro-inflammatory responses are likely to be evolutionarily programmed to resist fatal infections with an increased resistance to pathogens. Thus, inflammatory genotypes are an important and necessary part of the normal host responses to pathogens in early life, but the overproduction of inflammatory molecules might also cause immune-related inflammatory diseases and eventually death later. Therefore, low responder genotypes might better control inflammatory responses and age-related disease development, resulting in an increased chance of long life survival in a facilitory environment with reduced pathogen load and medical care, such as might be present in Western societies [12,14-16].

## Clonotypic immunity in ageing

Recently, longitudinal studies have shown that a cluster of immunological parameters can be used to evaluate the expectation and quality of life, i.e. the immunological risk phenotype [17,18]. Senescence of clonotypic immunity is most likely principally a result of alterations to T cells. Lifelong and chronic antigen load seems to be the major driving force of immunosenescence, which impacts on human lifespan by reducing the number of virgin antigen-non experienced T cells, and, simultaneously, fills the immunological space with expanded clones of memory and effector, antigen-experienced T cells [18-20]. Gradually, the T cell population shifts to a lower ratio of naïve cells to memory cells, the thymus pumps out fewer naïve T cells with age and those T cells remaining, especially the CD8+ subset, also show increased oligloclonality with age [4]. Thus, the repertoire of cells available to respond to antigenic challenge from previously unencountered pathogens is reduced. In addition, older organisms are often overrun by memory cells that carry a single type of T cell repertoire, i.e. clonal expansion. Thus, the memory cells from old individuals might recognize a limited set of antigens despite being plentiful in number. Many of the clonal expansions crowding an elderly person's immune system result from previous infections by persisting viruses [18,19].

In contrast to T cells, no evidence for a loss of B cell function has been found as neither the total number of B cells or immunoglobulin secreting cells have been shown to be profoundly decreased with age. However, the B-cell repertoire is influenced by ageing during an actual immune response, where the spectrum of expressed immunoglobulin genes, as well as the frequency of somatic mutations, affects the quality, though not necessarily the quantity, of the antibody response, which is highly relevant in clinical practice [7,21].

## *Immunity & Ageing*. Why do we need an Open Access journal?

Considering the paramount function of the immune system during ageing, journals oriented towards gerontology and geriatric sciences are now publishing an increasing number of articles dealing with immunology and ageing, but a specialised journal in this area does not exist. *Immunity & Ageing *was conceived to cover all topics relevant to immunity and ageing in an interdisciplinary manner. It is clear that immunological mechanisms are involved in process and manifestation of ageing in many systems. An example is displayed by the increase in serum and tissue levels of circulation pro-inflammatory cytokines which is accompanied by typical cellular ageing phenomena such as telomeric loss, oxidative damage, DNA defects, accumulation of advanced glycosylation products, cellular loss and others. These defects may be stimulators of cytokine secretion, and subsequently, cytokines are released into the systemic circulation. This results in a slowly progressive endo-crinosenescence and neurosenescence. The different factors may influence each other in form of a vicious spiral [22].

The journal will provide an opportunity to focus the topic of immunology of ageing, which is emerging as one of the critical mechanisms of ageing which aspects should get to all scientists physicians and other professions who are involved in the different inter-related disciplines, and the bridge that is emerging between these fields.

Furthermore, as an online, Open Access journal, *Immunity & Ageing *will promote "immediate" accessibility to this fast moving area of research, which is generally not possible for articles published in printed journals. From a modern perspective, electronic publishing is obviously attractive for its speed, easy global access and low cost, which all present considerable advantages over print and are seen as attractive factors by authors, readers and publishers. Open Access appears to provide exactly what people would like: rapid publication for authors, free access to information for readers, and inexpensive, global availability to readers for publishers [23]. We believe knowledge should belong to those who want it and access should not be unjustly and unacceptably expensive or difficult. *Immunity & Ageing *will take advantage of the Open Access policy to make peer-reviewed information widely and almost immediately available. We hope this forum, focussing on the themes of ageing and immunology, will create a new perspective for looking at a world that is inevitably becoming older. Creating and pushing forward a research knowledge base in *Immunity & Ageing *provides a unique opportunity to dissect out some parameters which might make health span equate with increasing lifespan.
